# Attempts to Access a Series of Pyrazoles Lead to New Hydrazones with Antifungal Potential against *Candida* species including Azole-Resistant Strains

**DOI:** 10.3390/molecules26195861

**Published:** 2021-09-27

**Authors:** Georgiana Negru, Laure Kamus, Elena Bîcu, Sergiu Shova, Boualem Sendid, Faustine Dubar, Alina Ghinet

**Affiliations:** 1 Faculty of Chemistry, “Alexandru Ioan Cuza” University of Iași, Bd. Carol I 11, 700506 Iași, Romania; georgiana_16_95@yahoo.com; 2 Inserm U1285, CHU Lille, UGSF UMR CNRS 8576, Glycobiology in Fungal and Clinical Applications, Unité de Glycobiologie Structurale et Fonctionnelle, University of Lille, 59000 Lille, France; laurekamus@aol.com (L.K.); boualem.sendid@univ-lille.fr (B.S.); 3 “Petru Poni” Institute of Macromolecular Chemistry, 700487 Iași, Romania; shova@icmpp.ro; 4 Laboratory of Sustainable Chemistry and Health, Health and Environment Department, Junia, 59000 Lille, France; 5 Inserm, CHU Lille, Institut Pasteur Lille, U1167—RID-AGE—Facteurs de Risque et Déterminants Moléculaires des Maladies Liées au Vieillissement, University of Lille, 59000 Lille, France

**Keywords:** hydrazone, *Candida* species, antifungal agents, *Candida albicans*, *Candida glabrata*

## Abstract

The treatment of benzylidenemalononitriles with phenylhydrazines in refluxing ethanol did not provide pyrazole derivatives, but instead furnished hydrazones. The structure of hydrazones was secured by X-ray analysis. The chemical proof was also obtained by direct reaction of 3,4,5-trimethoxybenzaldehyde with 2,4-dichlorophenylhydrazine. Newly synthesized hydrazones were tested against eight *Candida* spp. strains in a dose response assay to determine the minimum inhibitory concentration (MIC_99_). Five compounds were identified as promising antifungal agents against *Candida* spp. (*C. albicans* SC5314, *C. glabrata*, *C. tropicalis*, *C. parapsilosis* and *C. glabrata* (R azoles)), with MIC_99_ values ranging from 16 to 32 µg/mL and selective antifungal activity over cytotoxicity.

## 1. Introduction

Antifungal therapies evolved slowly during the early years of the twentieth century, with the development of antifungal agents lagging that of antibacterial agents. The current therapeutic arsenal for the systemic treatment of antifungal infections mainly includes polyenes, azoles, echinocandins and pyrimidine classes of compounds.

In 2020, the World Health Organization (WHO) set up the “WHO antifungal expert group on identifying priority fungal pathogens” posing a high risk to human health [1]. The azole-resistant fungal pathogens *Candida* spp. were identified as preoccupying pathogens of global public health importance. The main *Candida* species resistant to azoles drugs is *Candida krusei* (intrinsic resistance), while those with an acquired resistance are certain strains of *Candida glabrata* and rare strains of *Candida tropicalis*, *Candida parapsilosis* and *Candida kefyr*. The lack of therapeutic innovations or new chemical families under development for the discovery of therapeutic alternatives has reached a critical level.

Identifying new experimental drugs is challenging and expected more than ever. There are only a few different classes from a mechanistic point of view that are currently used to treat serious fungal infections.

The treatment of invasive candidiasis, which represent the most serious clinical forms in hospitals, is the subject of several international recommendations, in particular those published by the Infectious Diseases Society of America (IDSA) [2] and the European Society of Clinical Microbiology and Infectious Diseases (ESCMID) [3,4,5], with some adaptations dependent on involved *Candida* species and hospital wards (resuscitation, oncohematology, neonatology, etc.).

Currently, treatment is based on the use of fluconazole, if the patient is in stable condition, or on the use of echinocandins, if the patient is in a severe state. Fluconazole and caspofungin are consequently the most used drugs in clinics today for the treatment of *Candida* infections. Fluconazole, a fungistatic from the azole family, diminishes the conversion of lanosterol to ergosterol, which affects fungal membrane functions and inhibits cell multiplication. Caspofungin, from the family of echinocandins, has fungicidal activity against *Candida* spp. through inhibition of the synthesis of β-(1,3)-d-glucan, a major constituent of the fungal cell wall [6].

In case of intolerance to these molecules or resistance demonstrated by antifungal susceptibility testing (AST), liposomal amphotericin B is considered as a good alternative choice.

The main limitations of the use of these molecules are related to: (i) the pharmacokinetic features of each molecule; (ii) susceptibility to antifungals of the *Candida* species involved in tissue infection; (iii) the clinical background of the treated patient (neutropenic or not, neonatology, solid organ transplant, etc.).

**Figure 1:**
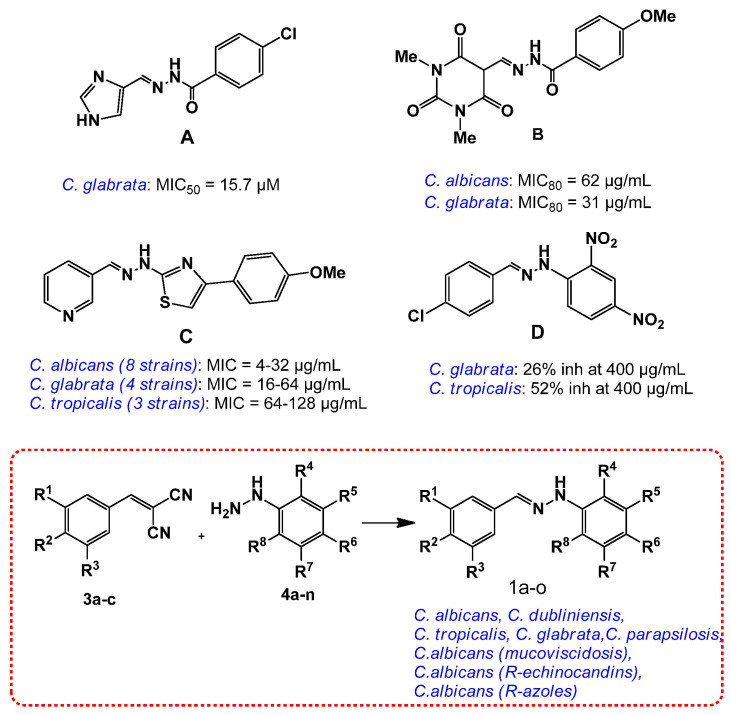
Structure of a selection of anti-Candida experimental drugs (**A**–**D**) and of target hydrazones **1a**–**o**.

Hydrazone derivatives and especially acylhydrazones have been described as antifungal agents on *Candida* spp. Acylhydrazone **A** (Figure 1) displayed antifungal potential on *C. glabrata* [7]. Acylbarbituric acid hydrazone **B** inhibited the growth of *C. albicans* and *C. glabrata* with MIC_80_ values of 62 µg/mL and 31 µg/mL, respectively (Figure 1) [8]. Hydrazone **C** was assayed for broad-spectrum antifungal activity against clinical isolates of *Candida* spp. and showed bioactivity against *C. albicans*, *C. glabrata* and *C. tropicalis,* with MIC values ranging from 4 to 128 µg/mL (Figure 1) [9]. Moderate antifungal potency was also registered for hydrazone **D**, bearing electro-withdrawing substituents on *C. glabrata* and *C. tropicalis* (Figure 1) [10]. In this report, a new series of hydrazones was synthesized and identified as effective on *Candida* spp., including azole- or echinocandin-resistant strains (compounds **1a**–**o**, Figure 1).

## 2. Results and Discussion

**Scheme 1:**
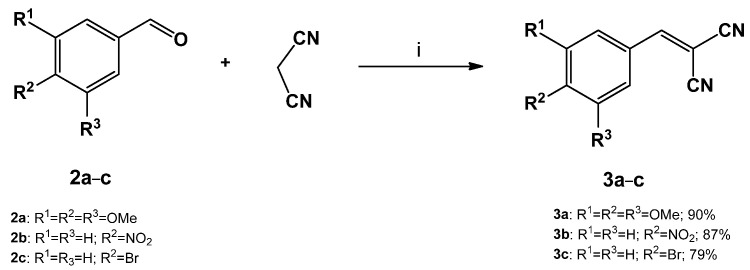
Reagents and conditions: (i) piperidine, EtOH, reflux, 6–8 h.

**Scheme 2:**
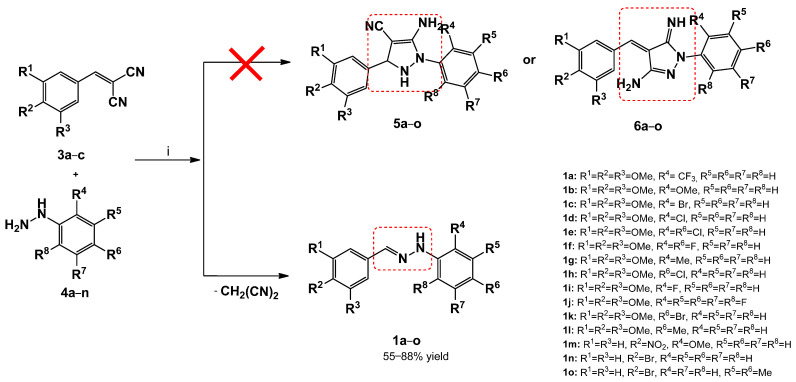
Reagents and conditions: (i) EtOH, reflux, 4–8 h.

**Figure 2:**
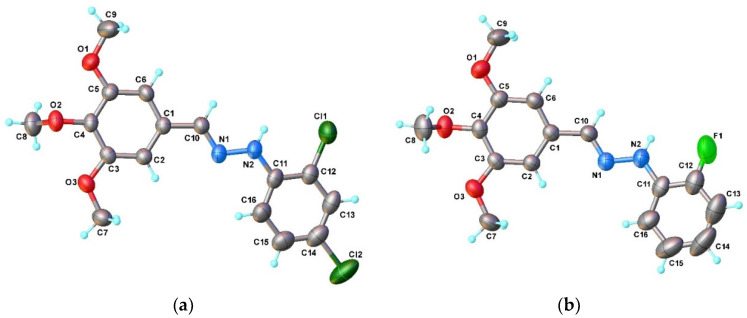
A view of the asymmetric part in the crystal structure of **1e** (**a**) and **1i** (**b**) with atom labeling and thermal ellipsoids at 50% level.

**Scheme 3:**
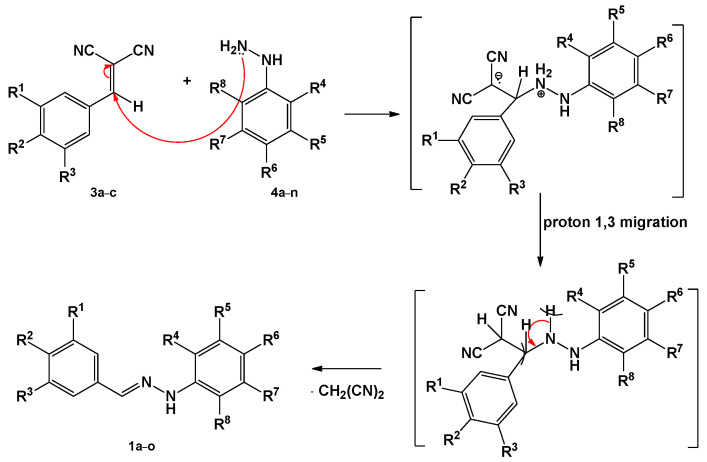
Proposed mechanism for the formation of hydrazones **1a**–**o** from benzylidenemalononitriles **3a**–**c** upon reaction with hydrazines **4a**–**n**.

In the frame of an ongoing medicinal chemistry program, an attempt to access new pyrazole derivatives of general structures **5** and **6**, starting from benzylidenemalononitriles **3a**–**c**, has been tried. The latter have been easily obtained by the Knoevenagel reaction of aldehydes **2a**–**c** with malononitrile in the presence of piperidine in refluxing ethanol (Scheme 1 and Scheme 2). They were next reacted with substituted phenylhydrazines **4a**–**n** in ethanol at reflux. The expected pyrazolines **5a**–**o** and/or **6a**–**o** could not be obtained. The same operatory conditions were previously described to afford aminopyrazole derivatives [11,12]. However, in our case, no trace of the target heterocycle was detected in the crude by ^1^H-NMR and TLC monitoring. Instead, in all cases, a different product was detected and isolated. The structure of the final product was resolved as a hydrazone derivative and secured by performing an X-ray on compounds **1e** and **1i** of the series (Figure 2). In addition, a chemical proof was also obtained by reacting aldehyde **2a** with hydrazine **4e,** which provided the same hydrazone **1e**. This confirmed the loss of the malononitrile unit during the reaction of benzylidenemalononitriles **3a**–**c** with phenylhydrazines **4a**–**n**, explaining undoubtedly the formation of hydrazones **1a**–**o** (Scheme 3).

The structure of compounds **1e** and **1i** was demonstrated by single crystal X-ray diffraction method. According to X-ray crystallography, the two compounds are isostructural. They crystallize in the *P*2_1_/*c* space group with close unit cell parameters (Table S1, see Supplementary Materials for full data). The asymmetric part of the unit cell (Figure 2a,b) comprises one molecule of **1e** and **1i** as a crystallographic independent unit, respectively. There are no co-crystallized solvent molecules in both crystals.

**Figure 3:**
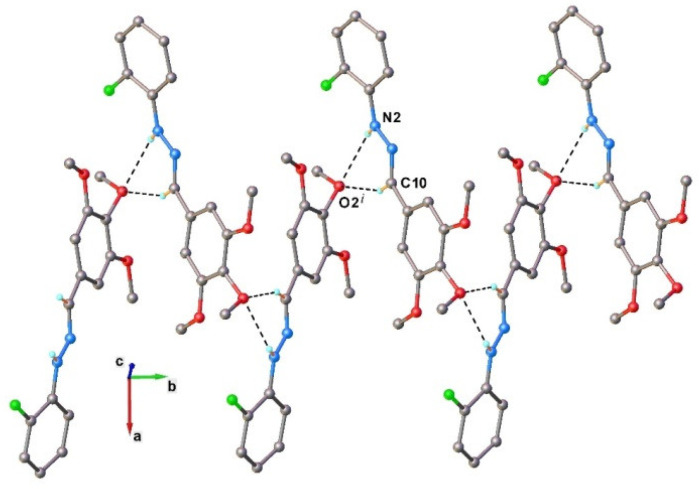
1D supramolecular chain in the crystal structure of **1i**. Non-relevant hydrogen atoms are not shown. H-bonds are drawn as black-dashed lines. Symmetry code: *i*) −*x*, −0.5 + *y*, −1.5 − *z*. H-bond parameters: N2-H···O2 [N2-H 0.86 Å, H···O2 2.65 Å, N2···O2′ 3.432(2) Å, ∠N2HO2 151.4°]; C10-H···O2 [C10-H 0.93 Å, H···O2 2.59 Å, C10···O2′ 3.470(2) Å, ∠C10HO2 158.4°].

As expected, compounds **1e** and **1i** feature similar crystal structure packing. Indeed, for both crystals, the main crystal structure motif is described as a one-dimensional supramolecular array running along the *b* axis, which is formed through intermolecular NH···O and CH···O hydrogen bonding. As an example, a view of the one-dimensional architecture in the crystal structure of **1i** is shown in Figure 3.

A mechanism has been proposed for the formation of hydrazones **1a**–**o** from benzylidenemalononitriles **3a**–**c** (Scheme 3). The first step involved the classical nucleophilic attack of the marginal nitrogen of the hydrazine on the ethylenic carbon of the 2-cyano-3-aryl-acrylonitrile. The intermediate formed underwent a proton 1,3 migration. This allowed the formation of malononitrile as a leaving group and the formation of hydrazones (Scheme 2 and Scheme 3).

Primary antifungal screening study by whole cell growth inhibition assays, using all the synthesized hydrazones **1a**–**o** at a single concentration of 32 µg/mL, was realized in triplicate (*n* = 3). Hit confirmation of active compounds by whole cell growth inhibition assays was conducted as an eight-point dose response to determine the Minimum Inhibitory Concentration (MIC), in triplicate (*n* = 3). The inhibition of growth was measured against eight fungi strains: *C. albicans* SC5314, *C. dubliniensis*, *C. glabrata*, *C. parapsilosis*, *C. albicans* from mucoviscidosis patients (*C. albicans* (mucoviscidosis)), *C. albicans* resistant to echinocandins (*C. albicans* (R echinocandins)) and *C. glabrata* resistant to azoles (*C. glabrata* (R azoles)) (Table 1). The fungal strains were obtained from Pôle de Biologie Pathologie Génétique, Centre Hospitalier Universitaire (CHU) de Lille, France. Fluconazole was used as the positive reference in the assay. Samples were prepared in DMSO and water to a final testing concentration of 32 µg/mL and in triplicate (*n* = 3), keeping the final DMSO concentration to a maximum of 1% DMSO. All the sample preparation was performed using liquid handling robots. Only five hydrazones, **1c**, **1d**, **1i**, **1k** and **1l,** displayed notable antifungal activity against tested *Candida* spp., with MIC values between 16 and 32 µg/mL. The results are presented in Table 1. All other synthesized hydrazones, **1a**, **1b**, **1e–h**, **1j** and **1m–o**, were less active, with MIC values > 32 µg/mL (data not shown).

Active hydrazones **1c**, **1d**, **1i**, **1k** and **1l** share the same 3,4,5-trimethoxyphenyl unit and have the particularity of a monosubstitution on the other phenyl ring. Generally, the *ortho*-substitution by an electro-withdrawing group (F, Cl and Br) in **1i**, **1d** and **1c**, respectively, was the most favorable chemical modulation for the antifungal activity in the current work. The comparison of halogens reveals that the chlorine atom in hydrazone **1d** was the most active, especially on *C. glabrata* (MIC = 16 µg/mL) compared to fluoro and bromo congeners **1i** and **1c** (MIC = 32 µg/mL) (Table 1). The trifluoromethyl substituent in hydrazone **1a** resulted in dramatical loss of the antifungal potential (MIC > 32 µg/mL). The *para*-bromo substitution in hydrazone **1k** slightly decreased the antifungal potential on *C. glabrata* (R azoles) (MIC > 32 µg/mL) but conserved the notable antifungal effect on *C. glabrata* (MIC = 16 µg/mL) and on *C. tropicalis* (MIC = 32 µg/mL) (Table 1). To be noted, only *para*-substituted compounds **1k** and **1l** displayed inhibition activity against *C. tropicalis* (MIC = 32 µg/mL), while the *ortho*-substituted analogs were less active (MIC > 32 µg/mL) (Table 1). The electron-donating substituents by inductive or mesomeric effect (Me or OMe) in the *ortho* position of the phenyl unit were not tolerated on any of the tested fungi (MIC > 32 µg/mL for hydrazones **1b**, **1g** and **1m**). The di- or polysubstitution of the same phenyl ring by both electron-withdrawing or electron-donating groups in hydrazones **1e**, **1f**, **1j** and **1o** were not favorable for antifungal activity (MIC > 32 µg/mL). Finally, the replacement of the 3,4,5-trimethoxyphenyl unit by a 4-nitrophenyl or 4-bromophenyl moiety in hydrazones **1m**–**o** also abolished the antifungal activity against all tested *Candida* spp. (MIC > 32 µg/mL). The 3,4,5-trimethoxyphenyl unit seemed essential to maintain the biological activity on *C. albicans* SC5314, *C. glabrata*, *C. parapsilosis* and *C. glabrata* resistant to azoles (MIC values of 16 and 32 µg/mL, respectively). Diminished potential was registered on the clinical isolates of *C. albicans* (mucoviscidosis) and *C. albicans* resistant to echinocandins. Newly synthesized hydrazones generally showed more pronounced antifungal activity on strains of *C. glabrata,* including *C. glabrata* that is resistant to azoles (Table 1).

**Figure 4:**
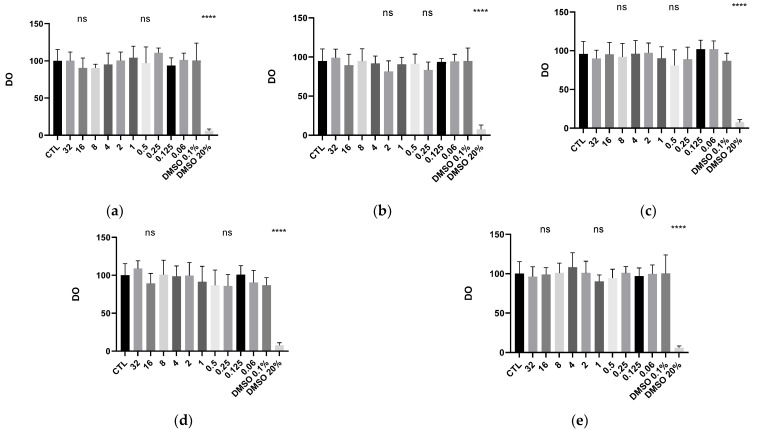
Evaluation of the cytotoxicity of experimental antifungals on HEK293 cells at different concentrations (32 µg/mL to 0.06 µg/mL): (**a**) hydrazone **1c**; (**b**) hydrazone **1d**; (**c**) hydrazone **1i**; (**d**) hydrazone **1k**; (**e**) hydrazone **1l**. ns: no significance; ****: *p* value ≤ 0.0001.

To verify the mammalian cytotoxicity of the newly identified antifungals presented herein, compounds **1c**, **1d**, **1i**, **1k** and **1l** were tested against human embryonic kidney cells (HEK293) at ten different concentrations (0.06 to 32 µg/mL) (Figure 4). The highest concentration of 32 µg/mL tested corresponded to the concentration at which the compounds exhibit potent antifungal activity. Since compounds were dissolved in 0.1% DMSO in the stock solution for this assay, DMSO was used as a negative reference in the same test. As depicted in Figure 4, the concentration of 0.1% of DMSO is devoid of cytotoxic effect and is safe for compound solubilization, while the concentration of 20% of DMSO displayed high toxicity. This denotes the importance of the concentration of DMSO used to dissolve the experimental drugs so as not to have distorted effects due to the solvent. All tested compounds showed no toxicity in viable kidney HEK293 cells.

In addition, compounds **1c**, **1d**, **1i**, **1k** and **1l** have also been selected and evaluated for cell growth inhibition activity on the NCI-60 cancer cell lines panel. Molecules were tested at 3.65 µg/mL (10 µM) concentration (compound **1c**), 3.21 µg/mL (10 µM) concentration (compound **1d**), 3.04 µg/mL (10 µM) concentration (compound **1i**), 3.65 µg/mL (10 µM) concentration (compound **1i**) and 3.00 µg/mL (10 µM) concentration (compound **1k**) and did not show any notable cytotoxic effect. The full one-dose mean graphs for antifungal agents **1c**, **1d**, **1i**, **1k** and **1l** are available in the supplementary data section associated with this article.

## 3. Materials and Methods

Starting materials are commercially available and were used without further purification (suppliers: Carlo Erba Reagents S.A.S., Val-de-Reuil, France; Thermo Fisher Scientific Inc., Illkirch, France; Tokyo Chemical Industry Co., Ltd., Zwijndrecht, Belgium; and Sigma-Aldrich Co., Saint-Quentin-Fallavier, France). Melting points were measured on an MPA 100 OptiMelt^®^ apparatus (Stanford Research Systems, Sunnyvale, CA, USA) and are uncorrected. Nuclear magnetic resonance (NMR) spectra were acquired at 400 MHz for ^1^H-NMR, and at 100 MHz for ^13^C-NMR on a Varian 400-MR spectrometer (Varian, Les Ulis, France) with tetramethylsilane (TMS) as internal standard, at room temperature (RT) or at 500 MHz for ^1^H-NMR, and at 125 MHz for ^13^C-NMR on a Bruker Avance III 500 MHz spectrometer (Bruker, Mannheim, Germany) with tetramethylsilane (TMS) as internal standard, at room temperature (RT). Chemical shifts (δ) are expressed in ppm relative to TMS. Splitting patterns are designed: s, singlet; d, doublet; dd, doublet of doublets; t, triplet; q, quadruplet; quint, quintuplet; m, multiplet; sym m, symmetric multiplet; br s, broaden singlet; br t, broaden triplet. Coupling constants (*J*) are reported in Hertz (Hz). The designation Ph1 in the ^13^C-NMR spectra description corresponds to the phenyl ring coming from the starting hydrazine, and Ph2 is the phenyl ring linked to -CH=N- group. Thin layer chromatography (TLC) was realized on Macherey Nagel silica gel plates (Macherey Nagel, Hoerdt, France) with fluorescent indicator and were visualized under a UV-lamp at 254 nm and 365 nm. Elemental analyses (C, H, N) of new compounds were determined on a Thermo Electron apparatus (Thermo Fisher Scientific Inc., Illkirch, France) by “Pôle Chimie Moléculaire-Welience”, Faculté des Sciences Mirande, Dijon, France.

### 3.1. General Procedure for the Synthesis of Benzylidenemalononitriles (***3a**–**c***)

A mixture of aldehyde (**2a**–**c**, 5.4–25.5 mmol, 1 equiv), malononitrile (5.4–25.5 mmol, 1 equiv) and 0.5 mL of piperidine in ethanol, was stirred at reflux for 6–8 h. After cooling the reaction medium to room temperature, the obtained precipitate was filtered, washed with ethanol, and then recrystallized from ethanol to afford the pure expected product (**3a**–**c**).

#### 3.1.1. 2-(3,4,5-trimethoxybenzylidene)malononitrile (**3a**)

The general procedure was used with 3,4,5-trimethoxybenzaldehyde (5.0 g) and malononitrile (1.4 mL) to obtain pure compound **3a** as a yellow solid (5.6 g, 90% yield); mp (EtOH) 119–121 °C (lit. mp 120 °C) [13]; R_f_ (EtOAc:Cyclohexane 1:1) = 0.84; ^1^H-NMR (CDCl_3_, 500 MHz) δ ppm 3.90 (s, 6H, 2OC*H*_3_), 3.97 (s, 3H, OC*H*_3_), 7.18 (s, 2H, 2Ar*H*), 7.65 (s, 1H, =C*H*). Compound **3a** exhibited the same NMR spectra as previously reported [13].

#### 3.1.2. 2-(4-nitrobenzylidene)malononitrile (**3b**)

The general procedure was used with 4-nitrobenzaldehyde (1.0 g) and malononitrile (0.37 mL) to obtain pure compound **3b** as a brown solid (1.04 g, 79% yield); mp (EtOH) 160–162 °C; R_f_ (EtOAc:Cyclohexane 1:1) = 0.83. ^1^H-NMR (CDCl_3_, 400 MHz) δ ppm 7.88 (s, 1H, =C*H*), 8.08 (d, *J* = 8.8 Hz, 2H, 2Ar*H*), 8.39 (d, *J* = 8.8 Hz, 2H, 2Ar*H*). Compound **3c** exhibited the same NMR spectra as previously reported [14].

#### 3.1.3. 2-(4-bromobenzylidene)malononitrile (**3c**)

The general procedure was used with 4-bromobenzaldehyde (1.0 g) and malononitrile (0.3 mL) to obtain pure compound **3c** as a white solid (1.1 g, 87% yield); mp (EtOH) 162–164 °C (lit. mp 165–166 °C) [15]; R_f_ (EtOAc:Cyclohexane 1:1) = 0.93. ^1^H-NMR (DMSO-*d_6_*, 500 MHz) δ ppm 7.81–7.86 (m, 4H, 4Ar*H*), 8.51 (s, 1H, =C*H*). Compound **3c** exhibited the same NMR spectra as previously reported [15].

### 3.2. General Procedure for the Preparation of Hydrazone Derivatives (***1a**–**o***)

A solution of benzylidenemalononitrile (**3a**–**c**, 1.6–2.5 mmol, 1 equiv.) and hydrazine (**4a**–**n**, 1.6–2.5 mmol, 1 equiv.) in ethanol was stirred at reflux for 4–8 h. After cooling the reaction medium to room temperature, the product precipitated was collected by filtration, washed with ethanol and purified by recrystallization from ethanol to obtain pure target hydrazone.

#### 3.2.1. 1-(2-(trifluoromethyl)phenyl)-2-(3,4,5-trimethoxybenzylidene)hydrazine (**1a**)

The general procedure was used with 2-(3,4,5-trimethoxybenzylidene)malononitrile 3a (0.4 g) and 2-(trifluoromethyl)phenylhydrazine **4a** (0.29 g) to obtain pure compound **1a** as a white solid (0.46 g, 79% yield); mp (EtOH) 147–148 °C; R_f_ (EtOAc:Cyclohexane 1:1) = 0.64. ^1^H-NMR (CDCl_3_, 500 MHz) δ ppm 3.89 (s, 3H, OC*H*_3_), 3.92 (s, 6H, 2OC*H*_3_), 6.89–6.92 (m, 3H, 3Ar*H*), 7.47–7.50 (m, 2H, 2Ar*H*), 7.73 (s, 1H, =C*H*), 7.78 (d, *J* = 9.0 Hz, 1H, Ar*H*), 8.01 (s, 1H, N*H*). ^13^C-NMR (CDCl_3_, 125 MHz) δ ppm 56.3 (2OC*H*_3_), 61.1 (OC*H*_3_), 103.7 (2CH, 2CH-2,6-Ph2), 112.3 (q, *J* = 60.0, 30.0 Hz, CF_3_), 114.8 (CH, CH-6-Ph1), 119.1 (CH, CH-5-Ph1), 125.0 (q, *J* = 541.2, 270.0 Hz, C, C-2-Ph1), 126.3 (q, *J* = 11.2, 6.2 Hz, CH, CH-3-Ph1), 130.4 (C, C-1-Ph1), 133.3 (CH, CH-4-Ph1), 140.1 (=C*H*), 142.2 (C, C-4-Ph2), 142.3 (C, C-1-Ph2), 153.6 (2C, 2C-3,5-Ph2). Elemental analysis calcd (%) for C_17_H_17_F_3_N_2_O_3_: C 57.63, H 4.84, N 7.91; found: C 57.92, H 4.89, N 7.33.

#### 3.2.2. 1-(2-methoxyphenyl)-2-(3,4,5-trimethoxybenzylidene)hydrazine (**1b**)

The general procedure was used with 2-(3,4,5-trimethoxybenzylidene)malononitrile **3a** (0.4 g) and 2-methoxyphenylhydrazine **4b** (0.23 g) to obtain pure compound **1b** as a yellow solid (0.37 g, 71% yield); mp (EtOH) 179–180 °C; R_f_ (EtOAc:Cyclohexane 1:1) = 0.66. ^1^H-NMR (CDCl_3_, 500 MHz) δ ppm 3.88 (s, 3H, OC*H*_3_), 3.89 (s, 3H, OC*H*_3_), 3.92 (s, 6H, 2OC*H*_3_), 6.83–6.86 (m, 2H, 2Ar*H*), 6.91 (s, 2H, 2Ar*H*), 6.98 (td, *J* = 7.0, 2.0 Hz, 1H, Ar*H*), 7.52 (dd, *J* = 7.5, 2.0 Hz, 1H, Ar*H*), 7.70 (s, 1H, =C*H*), 8.07 (s, 1H, N*H*). ^13^C-NMR (CDCl_3_, 125 MHz) δ ppm 55.7 (OC*H*_3,_ OC*H*_3_-2-Ph1), 56.3 (2OC*H*_3_), 61.1 (OC*H*_3_), 103.3 (2CH, 2CH-2,6-Ph2), 110.2 (CH, CH-6-Ph1), 112.5 (CH, CH-3-Ph1), 119.4 (CH, CH-4-Ph1), 121.7 (CH, CH-5-Ph1), 131.2 (C, C-1-Ph2), 134.3 (C, C-1-Ph1), 138.1 (=C*H*), 138.7 (C, C-2-Ph1), 145.3 (C, C-4-Ph2), 153.6 (2C, 2C-3,5-Ph2). Elemental analysis calcd (%) for C_17_H_20_N_2_O_4_: C 64.54, H 6.37, N 8.86; found: C 64.87, H 6.52, N 9.02.

#### 3.2.3. 1-(2-bromophenyl)-2-(3,4,5-trimethoxybenzylidene)hydrazine (**1c**)

The general procedure was used with 2-(3,4,5-trimethoxybenzylidene)malononitrile **3a** (0.4 g) and 2-bromophenylhydrazine **4c** (0.31 g) to obtain pure compound **1c** as a pink solid (0.50 g, 83% yield); mp (EtOH) 155–157 °C; R_f_ (EtOAc:Cyclohexane 1:1) = 0.65. ^1^H-NMR (CDCl_3_, 500 MHz) δ ppm 3.89 (s, 3H, OC*H*_3_), 3.92 (s, 6H, 2OC*H*_3_), 6.74 (td, *J* = 8.0, 1.5 Hz, 1H, Ar*H*), 6.91 (s, 2H, 2Ar*H*), 7.28 (td, *J* = 8.0, 1.5 Hz, 1H, Ar*H*), 7.44 (dd, *J* = 8, 1.5 Hz, 1H, Ar*H*), 7.58 (dd, *J* = 8.0, 1.5 Hz, 1H, Ar*H*), 7.76 (s, 1H, =C*H*), 8.05 (s, 1H, N*H*). ^13^C-NMR (CDCl_3_, 125 MHz) δ ppm 56.3 (2OC*H*_3_), 61.1 (OC*H*_3_), 103.5 (2CH, 2CH-2,6-Ph2), 106.9 (C, C-2-Ph1), 114.5 (CH, CH-6-Ph1), 120.7 (CH, CH-4-Ph1), 128.7 (CH, CH-5-Ph1), 130.6 (C, C-1-Ph2), 132.4 (CH, CH-3-Ph1), 139.0 (C, C-4-Ph2), 139.6 (=C*H*), 141.5 (C, C-1-Ph1), 153.6 (2C, 2C-3,5-Ph2). Elemental analysis calcd (%) for C_16_H_17_BrN_2_O_3_: C 52.62, H 4.69, N 7.67; found: C 52.76, H 4.90, N 7.95.

#### 3.2.4. 1-(2-chlorophenyl)-2-(3,4,5-trimethoxybenzylidene)hydrazine (**1d**)

The general procedure was used with 2-(3,4,5-trimethoxybenzylidene)malononitrile **3a** (0.4 g) and 2-chlorophenylhydrazine **4d** (0.23 g) to obtain pure compound **1d** as a pink solid (0.37 g, 71% yield); mp (EtOH) 164–166 °C; R_f_ (EtOAc:Cyclohexane 1:1) = 0.66. ^1^H-NMR (CDCl_3_, 500 MHz) δ ppm 3.86 (s, 3H, OC*H*_3_), 3.92 (s, 6H, 2OC*H*_3_), 6.80 (td, *J* = 8.0, 1.5 Hz, 1H, Ar*H*), 6.90 (s, 2H, 2Ar*H*), 7.23–7.29 (m, 2H, 2Ar*H*), 7.60 (dd, *J* = 8.0, 1.5 Hz, 1H, Ar*H*), 7.73 (s, 1H, =C*H*), 8.05 (s, 1H, N*H*). ^13^C-NMR (CDCl_3_, 125 MHz) δ ppm 56.2 (2OC*H*_3_), 61.1 (OC*H*_3_), 103.5 (2CH, 2CH-2,6-Ph2), 114.2 (CH, CH-6-Ph1), 117.0 (C, C-2-Ph1), 120.1 (CH, CH-5-Ph1), 128.0 (CH, CH-3-Ph1), 129.2 (CH, CH-4-Ph1), 130.6 (C, C-1-Ph2), 139.0 (C, C-4-Ph2), 139.6 (=C*H*), 140.6 (C, C-1-Ph1), 153.6 (2C, 2C-3,5-Ph2). Elemental analysis calcd (%) for C_16_H_17_ClN_2_O_3_: C 59.91, H 5.34, N 8.73; found: C 60.22, H 5.66, N 8.98.

#### 3.2.5. 1-(2,4-dichlorophenyl)-2-(3,4,5-trimethoxybenzylidene)hydrazine (**1e**)

The general procedure was used with 2-(3,4,5-trimethoxybenzylidene)malononitrile **3a** (0.4 g) and 2,4-dichlorophenylhydrazine **4e** (0.29 g) to obtain pure compound **1e** as a white solid (0.46 g, 79% yield); mp (EtOH) 185–187 °C; R_f_ (EtOAc:Cyclohexane 1:1) = 0.66. ^1^H-NMR (CDCl_3_, 500 MHz) δ ppm 3.88 (s, 3H, OC*H*_3_), 3.90 (s, 6H, 2OC*H*_3_), 6.87 (s, 2H, 2Ar*H*), 7.18 (dd, *J* = 8.5, 2.0 Hz, 1H, Ar*H*), 7.25 (d, *J* = 2.0 Hz, 1H, Ar*H*), 7.50 (d, *J* = 8.5 Hz, 1H, Ar*H*), 7.69 (s, 1H, =C*H*), 7.96 (s, 1H, N*H*). ^13^C-NMR (CDCl_3_, 125 MHz) δ ppm 56.2 (2OC*H*_3_), 61.0 (OC*H*_3_), 103.5 (2CH, 2CH-2,6-Ph2), 114.9 (CH, CH-6-Ph-1), 117.1 (C, C-2-Ph1), 124.0 (C, C-4-Ph1), 128.1 (CH, CH-5-Ph1), 128.7 (CH, CH-3-Ph1), 130.3 (C, C-1-Ph2), 139.1 (C, C-4-Ph2), 139.4 (C, C-1-Ph-1), 140.2 (=C*H*), 153.5 (2C, 2C-3,5-Ph2). Elemental analysis calcd (%) for C_16_H_16_Cl_2_N_2_O_3_: C 54.10, H 4.54, N 7.89; found: C 54.29, H 4.79, N 8.01.

#### 3.2.6. 1-(2,4-difluorophenyl)-2-(3,4,5-trimethoxybenzylidene)hydrazine (**1f**)

The general procedure was used with 2-(3,4,5-trimethoxybenzylidene)malononitrile **3a** (0.4 g) and 2,4-difluorophenylhydrazine **4f** (0.24 g) to obtain pure compound **1f** as a yellow solid (0.34 g, 65% yield); mp (EtOH) 185–188 °C; R_f_ (EtOAc:Cyclohexane 1:1) = 0.53. ^1^H-NMR (DMSO-*d_6_*, 500 MHz) δ ppm 3.69 (s, 3H, OC*H*_3_), 3.84 (s, 6H, 2OC*H*_3_), 6.95 (s, 2H, 2Ar*H*), 6.99 (td, *J* = 9.0, 2.0 Hz, 1H, Ar*H*), 7.18 (td, *J* = 9.0, 2.0 Hz, 1H, Ar*H*), 7.53 (td, *J* = 9.0, 2.0 Hz, 1H, Ar*H*), 8.03 (s, 1H, =C*H*), 10.18 (s, 1H, N*H*). ^13^C-NMR (DMSO-*d_6_*, 125 MHz) δ ppm 55.9 (2OC*H*_3_), 60.1 (OC*H*_3_), 103.1 (2CH, 2CH-2,6-Ph2), 103.8 (CH, CH-3-Ph1), 111.4 (dd, *J* = 21.2, 2.5 Hz, CH, CH-5-Ph1), 114.2 (q, *J* = 8.7, 5.0 Hz, CH, CH-6-Ph1), 130.5 (q, *J* = 10.0, 2.5 Hz, C, C-1-Ph1), 131.1 (C, C-1-Ph2), 138.0 (C, C-4-Ph2), 139.4 (=C*H*), 148.4 (dd, *J* = 240.0, 11.2 Hz, C, C-2-Ph1), 153.2 (2C, 2C-3,5-Ph2), 154.7 (dd, *J* = 235.0, 10.0 Hz, C, C-4-Ph1). Elemental analysis calcd (%) for C_16_H_16_F_2_N_2_O_3_: C 59.62, H 5.00, N 8.69; found: C 59.90, H 5.23, N 8.88.

#### 3.2.7. 1-(*o*-tolyl)-2-(3,4,5-trimethoxybenzylidene)hydrazine (**1g**)

The general procedure was used with 2-(3,4,5-trimethoxybenzylidene)malononitrile **3a** (0.4 g) and *o*-tolylhydrazine **4g** (0.20 g) to obtain pure compound **1g** as a yellow solid (0.43 g, 88% yield); mp (EtOH) 200–202 °C; R_f_ (EtOAc:Cyclohexane 1:1) = 0.63. ^1^H-NMR (DMSO-*d_6_*, 500 MHz) δ ppm 2.19 (s, 3H, C*H_3_*), 3.75 (s, 3H, OC*H*_3_), 3.84 (s, 6H, 2OC*H*_3_), 6.66 (t, *J* = 7.5 Hz, 1H, Ar*H*), 6.84 (s, 2H, 2Ar*H*), 6.97 (d, *J* = 7.5 Hz, 1H, Ar*H*), 7.07 (t, *J* = 7.5 Hz, 1H, Ar*H*), 7.41 (d, *J* = 7.5 Hz, 1H, Ar*H*), 7.90 (s, 1H, =C*H*), 8.85 (br s, 1H, N*H*). ^13^C-NMR (DMSO-*d_6_*, 125 MHz) δ ppm 17.1 (CH_3_), 55.5 (2OC*H*_3_), 60.1 (OC*H*_3_), 102.4 (2CH, 2CH-2,6-Ph2), 112.0 (CH, CH-6-Ph1), 118.6 (CH, CH-5-Ph1), 120.3 (C, C-2-Ph1), 126.4 (CH, CH-4-Ph1), 129.8 (CH, CH-3-Ph1), 131.2 (C, C-1-Ph2), 137.5 (C, C-4-Ph2), 137.6 (=C*H*), 142.7 (C, C-1-Ph1), 152.8 (2C, 2C-3,5-Ph2). Elemental analysis calcd (%) for C_17_H_20_N_2_O_3_: C 67.98, H 6.71, N 9.33; found: C 68.19, H 6.93, N 9.55.

#### 3.2.8. 1-(4-chlorophenyl)-2-(3,4,5-trimethoxybenzylidene)hydrazine (**1h**)

The general procedure was used with 2-(3,4,5-trimethoxybenzylidene)malononitrile **3a** (0.4 g) and 4-chlorophenylhydrazine **4h** (0.23 g) to obtain pure compound **1h** as a yellow solid (0.41 g, 79% yield); mp (EtOH) 156–158 °C; R_f_ (EtOAc:Cyclohexane 1:1) = 0.66. ^1^H-NMR (CDCl_3_, 500 MHz) δ ppm 3.88 (s, 3H, OC*H*_3_), 3.89 (s, 6H, 2OC*H*_3_), 6.85 (s, 2H, 2Ar*H*), 7.01 (d, *J* = 9.0 Hz, 2H, 2Ar*H*), 7.20 (d, *J* = 9.0 Hz, 2H, 2Ar*H*), 7.51 (s, 1H, =C*H*), 7.73 (br s, 1H, N*H*). ^13^C-NMR (CDCl_3_, 125 MHz) δ ppm 56.2 (2OC*H*_3_), 61.0 (OC*H*_3_), 103.3 (2CH, 2CH-2,6-Ph2), 113.9 (2CH, 2CH-2,6-Ph1), 124.5 (C, C-4-Ph1), 129.2 (2CH, 2CH-3,5-Ph1), 130.9 (C, C-1-Ph2), 137.9 (=C*H*), 138.7 (C, C-4-Ph2), 143.4 (C, C-1-Ph1), 153.5 (2C, 2C-3,5-Ph2). Elemental analysis calcd (%) for C_16_H_17_ClN_2_O_3_: C 59.91, H 5.34, N 8.73; found: C 60.26, H 5.59, N 9.01.

#### 3.2.9. 1-(2-fluorophenyl)-2-(3,4,5-trimethoxybenzylidene)hydrazine (**1i**)

The general procedure was used with 2-(3,4,5-trimethoxybenzylidene)malononitrile **3a** (0.4 g) and 2-fluorophenylhydrazine **4i** (0.21 g) to obtain pure compound **1i** as a pink solid (0.37 g, 75% yield); mp (EtOH) 145–147 °C; R_f_ (EtOAc:Cyclohexane 1:1) = 0.61. ^1^H NMR (CDCl_3_, 500 MHz) δ ppm 3.88 (s, 3H, OC*H*_3_), 3.91 (s, 6H, 2OC*H*_3_), 6.77–6.81 (m, 1H, Ar*H*), 6.89 (s, 2H, 2Ar*H*), 7.01–7.05 (m, 1H, Ar*H*), 7.11 (t, *J* = 8.0 Hz, 1H, Ar*H*), 7.59 (t, *J* = 8.0 Hz, 1H, Ar*H*), 7.66 (s, 1H, =C*H*), 7.79 (d, *J* = 2.5 Hz, 1H, N*H*). ^13^C-NMR (CDCl_3_, 125 MHz) δ ppm 56.2 (2OC*H*_3_), 61.0 (OC*H*_3_), 103.4 (2CH, 2CH-2,6-Ph2), 114.5 (d, *J* = 2.5 Hz, CH, CH-3-Ph1), 114.9 (d, *J* = 17.5 Hz, CH, CH-5-Ph1), 119.5 (d, *J* = 7.5 Hz, CH, CH-4-Ph1), 124.9 (d, *J* = 3.7 Hz, CH, CH-6-Ph1), 130.7 (C, C-1-Ph2), 133.1 (d, *J* = 8.7 Hz, C-1-Ph1), 138.9 (C, C-4-Ph2), 139.3 (=C*H*), 149.7 (d, *J* = 237.5 Hz, C, C-2-Ph1), 153.6 (2C, 2C-3,5-Ph2). Elemental analysis calcd (%) for C_16_H_17_FN_2_O_3_: C 63.15, H 5.63, N 9.21; found: C 63.40, H 5.89, N 9.39.

#### 3.2.10. 1-(pentafluorophenyl)-2-(3,4,5-trimethoxybenzylidene)hydrazine (**1j**)

The general procedure was used with 2-(3,4,5-trimethoxybenzylidene)malononitrile **3a** (0.4 g) and pentafluorophenylhydrazine **4j** (0.32 g) to obtain pure compound **1j** as an orange solid with the same NMR spectra as previously described [16] (0.47 g, 77% yield); mp (EtOH) 220–223 °C; R_f_ (EtOAc:Cyclohexane 1:1) = 0.45. ^1^H-NMR (DMSO-*d_6_*, 500 MHz) δ ppm 3.68 (s, 3H, OC*H*_3_), 3.80 (s, 6H, 3OC*H*_3_), 6.90 (s, 2H, 2Ar*H*), 8.01 (s, 1H, =C*H*), 10.28 (s, 1H, N*H*). ^13^C-NMR (DMSO-*d_6_*, 125 MHz) δ ppm 55.8 (2OC*H*_3_), 60.1 (OC*H*_3_), 103.2 (2CH, 2CH-2,6-Ph2), 121.3 (C, C-F), 130.4 (C, C-1-Ph2), 132.8 (C, C-F), 134.7 (C, C-F), 136.5 (C, C-F), 136.8 (C, C-F), 138.3 (C, C-1-Ph1), 138.8 (C, C-4-Ph2), 141.8 (=C*H*), 153.2 (2C, 2C-3,5-Ph2). Elemental analysis calcd (%) for C_16_H_13_F_5_N_2_O_3_: C 51.07, H 3.48, N 7.44; found: C 51.25, H 3.61, N 7.69.

#### 3.2.11. 1-(4-bromophenyl)-2-(3,4,5-trimethoxybenzylidene)hydrazine (**1k**)

The general procedure was used with 2-(3,4,5-trimethoxybenzylidene)malononitrile **3a** (0.4 g) and 4-bromophenylhydrazine **4k** (0.31 g) to obtain pure compound **1k** as a white solid (0.44 g, 73% yield); mp (EtOH) 152–155 °C; R_f_ (EtOAc:Cyclohexane 1:1) = 0.92. ^1^H-NMR (CDCl_3_, 500 MHz) δ ppm 3.88 (s, 3H, OC*H*_3_), 3.91 (s, 6H, 2OC*H*_3_), 6.87 (s, 2H, 2Ar*H*), 6.97 (d, *J* = 8.0 Hz, 2H, 2Ar*H*), 7.35 (d, *J* = 8.0 Hz, 2H, 2Ar*H*), 7.56 (s, 1H, =C*H*), 7.67 (s, 1H, N*H*). ^13^C-NMR (CDCl_3_, 125 MHz) δ ppm 56.2 (2OC*H*_3_), 61.0 (OC*H*_3_), 103.2 (2CH, 2CH-2,6-Ph2), 111.8 (C, C-4-Ph1), 114.3 (2CH, 2CH-2,6-Ph1), 130.7 (C, C-1-Ph2), 132.1 (2CH, 2CH-3,5-Ph1), 137.9 (=C*H*), 138.7 (C, C-4-Ph2), 143.7 (C, C-1-Ph1), 153.5 (2C, 2C-3,5-Ph2). Elemental analysis calcd (%) for C_16_H_17_BrN_2_O_3_: C 52.62, H 4.69, N 7.67; found: C 52.81, H 4.95, N 7.88.

#### 3.2.12. 1-(*p*-tolyl)-2-(3,4,5-trimethoxybenzylidene)hydrazine (**1l**)

The general procedure was used with 2-(3,4,5-trimethoxybenzylidene)malononitrile **3a** (0.4 g) and *p*-tolylhydrazine **4l** (0.20 g) to obtain pure compound **1l** as a yellow solid (0.27 g, 55% yield); mp (EtOH) 172–174 °C; R_f_ (EtOAc:Cyclohexane 1:1) = 0.82. ^1^H-NMR (CDCl_3_, 500 MHz) δ ppm 2.38 (s, 3H, C*H*_3_), 3.87 (s, 9H, 3OC*H*_3_), 5.53 (s, 1H, N*H*), 6.66 (s, 2H, 2Ar*H*), 7.20 (d, *J* = 8.0 Hz, 2H, 2Ar*H*), 7.48 (d, *J* = 8.0 Hz, 2H, 2Ar*H*). ^13^C-NMR (CDCl_3_, 125 MHz) δ ppm 21.5 (CH_3_), 56.2 (2OC*H*_3_), 60.9 (OC*H*_3_), 85.7 (=C*H*), 106.1 (2CH, 2CH-2,6-Ph2), 122.4 (2CH, 2CH-2,6-Ph1), 129.7 (2CH, 2CH-3,5-Ph1), 133.1 (C, C-1-Ph2), 137.4 (C, C-4-Ph1), 141.4 (C, C-4-Ph2), 150.3 (C, C-1-Ph1), 153.1 (2C, 2C-3,5-Ph2). Elemental analysis calcd (%) for C_17_H_20_N_2_O_3_: C 67.98, H 6.71, N 9.33; found: C 68.07, H 6.85, N 9.50.

#### 3.2.13. 1-(2-methoxyphenyl)-2-(4-nitrobenzylidene)hydrazine (**1m**)

The general procedure was used with 2-(4-nitrobenzylidene)malononitrile **3b** (0.5 g) and 2-methoxyphenylhydrazine **4b** (0.34 g) to obtain pure compound **1m** as a dark red solid (0.46 g, 68% yield); mp (EtOH) 165–167 °C; R_f_ (EtOAc:Cyclohexane 1:1) = 0.49. ^1^H-NMR (DMSO-*d_6_*, 500 MHz) δ ppm 3.86 (s, 3H, OC*H*_3_), 6.83 (t, *J* = 7.5 Hz, 1H, Ar*H*), 6.92 (t, *J* = 7.5 Hz, 1H, Ar*H*), 6.97 (d, *J* = 8.0 Hz, 1H, Ar*H*), 7.46 (dd, *J* = 8.0, 1.5 Hz, 1H, Ar*H*), 7.82 (dd, *J* = 9.0, 2.0 Hz, 2H, 2Ar*H*), 8.21 (dd, *J* = 9.0, 2.0 Hz, 3H, 2Ar*H* + =C*H*), 10.3 (s, 1H, N*H*). ^13^C-NMR (DMSO-*d_6_*, 125 MHz) δ ppm 55.6 (OC*H*_3_), 111.1 (CH, CH-3-Ph1), 112.3 (CH, CH-6-Ph1), 120.0 (CH, CH-4-Ph1), 121.3 (CH, CH-5-Ph1), 124.1 (2CH, 2CH-3,5-Ph2), 126.0 (2CH, 2CH-2,6-Ph2), 133.6 (C, C-1-Ph2), 135.2 (=C*H*), 142.7 (C, C-1-Ph1), 145.6 (C, C-4-Ph2), 146.0 (C, C-2-Ph1). Elemental analysis calcd (%) for C_14_H_13_N_3_O_3_: C 61.99, H 4.83, N 15.49; found: C 62.23, H 4.97, N 15.72.

#### 3.2.14. 1-(4-bromobenzylidene)-2-phenylhydrazine (**1n**)

The general procedure was used with 2-(4-bromobenzylidene)malononitrile **3c** (0.5 g) and phenylhydrazine **4m** (0.21 mL) to obtain pure compound **1n** as a white solid with the same NMR spectra as previously described [17] (0.36 g, 61% yield); mp (EtOH) 121–123 °C (lit. mp 116–117 °C) [17]; R_f_ (EtOAc:Cyclohexane 1:1) = 0.62. ^1^H-NMR (CDCl_3_, 500 MHz) δ ppm 6.88–6.91 (m, 1H, Ar*H*), 7.11 (dd, *J* = 9.0, 1.0 Hz, 2H, 2Ar*H*), 7.27–7.30 (m, 2H, 2Ar*H*), 7.48–7.53 (m, 5H, 4Ar*H* + N*H*), 7.61 (s, 1H, =C*H*). ^13^C-NMR (CDCl_3_, 125 MHz) δ ppm 112.9 (2CH, 2CH-3,5-Ph1), 120.5 (CH, CH-4-Ph1), 122.3 (C, C-4-Ph2), 127.7 (2CH, 2CH-2,6-Ph2), 129.5 (2CH, 2CH-2,6-Ph1), 131.9 (2CH, 2CH-3,5-Ph2), 134.4 (C, C-1-Ph2), 135.9 (=C*H*), 144.5 (C, C-1-Ph1). Elemental analysis calcd (%) for C_13_H_11_BrN_2_: C 56.75, H 4.03, N 10.18; found: C 56.93, H 4.34, N 10.33.

#### 3.2.15. 1-(4-bromobenzylidene)-2-(3,4-dimethylphenyl)hydrazine (**1o**)

The general procedure was used with 2-(4-bromobenzylidene)malononitrile **3c** (0.5 g) and 3,4-dimethylphenylhydrazine **4n** (0.29 g) to obtain pure compound **1o** as a white solid (0.45 g, 69% yield); mp (EtOH) 141–143 °C; R_f_ (EtOAc:Cyclohexane 1:1) = 0.59. ^1^H-NMR (CDCl_3_, 500 MHz) δ ppm 2.20 (s, 3H, C*H*_3_), 2.26 (s, 3H, C*H*_3_), 6.84 (d, *J* = 7.5 Hz, 1H, Ar*H*), 6.93 (s, 1H, Ar*H*), 7.03 (d,*J* = 8.5 Hz, 1H, Ar*H*), 7.46–7.54 (m, 4H, 4Ar*H*), 7.58 (br s, 2H, N*H* + =C*H*). ^13^C-NMR (CDCl_3_, 125 MHz) δ ppm 19.1 (CH_3_), 20.2 (CH_3_), 110.4 (CH, CH-5-Ph1), 114.4 (CH, CH-2-Ph1), 122.1 (C, C-4-Ph2), 127.6 (2CH, 2CH-2,6-Ph2), 128.6 (C, C-3-Ph1), 130.5 (CH, CH-6-Ph1), 131.9 (2CH, 2CH-3,5-Ph2), 134.6 (C, C-1-Ph2), 135.3 (=C*H*), 137.7 (C, C-4-Ph1), 142.6 (C, C-1-Ph1). Elemental analysis calcd (%) for C_15_H_15_BrN_2_: C 59.42, H 4.99, N 9.24; found: C 59.65, H 5.07, N 9.50.

### 3.3. X-ray Crystallography

X-ray diffraction measurements for **1e** and **1i** were carried out with a Rigaku Oxford Diffraction XCALIBUR E CCD diffractometer (Rigaku Europe SE, Frankfurt, Germany) equipped with graphite-monochromated MoKα radiation. The unit cell determination and data integration were carried out using the CrysAlis package of Oxford Diffraction (Rigaku Europe SE, Frankfurt, Germany) [18]. The structures were solved by intrinsic phasing using Olex2 [19] software with the SHELXT [20] structure solution program and refined by full-matrix least-squares on *F*^2^ with SHELXL-2015 [20], using an anisotropic model for non-hydrogen atoms. All H atoms attached to carbon were introduced in idealized positions (d_CH_ = 0.96 Å) using the riding model, with their isotropic displacement parameters fixed at 120% of their riding atom. The positions of H atoms for NH groups were determined from Fourier synthesis maps and verified through the hydrogen bonds parameters. Table S1 provides a summary of the crystallographic data together with refinement details for compounds. The geometric parameters are summarized in Table S2. The supplementary crystallographic data can be obtained free of charge via www.ccdc.cam.ac.uk/conts/retrieving.html (accessed on 19 July 2021) (or from the Cambridge Crystallographic Data Centre, 12 Union Road, Cambridge CB2 1EZ, UK; fax: (+44) 1223–336-033; or deposit@ccdc.ca.ac.uk).

### 3.4. MIC_99_ Determination Assays

MIC_99_ determination against *Candida* spp. was performed according to the standard culture microdilution method from the Clinical and Laboratory Standard Institute (CLSI). Inocula from *Candida* spp. strains were obtained from fungal cultures in Sabouraud dextrose agar (SDA) at 37 °C for 24 h. The initial concentration of *Candida* spp. strains was 1–5 × 10^6^ CFU/mL. The inocula were adjusted in order to obtain an optical density of 0.5 in the McFarland scale using sterile mQ water. Cells were suspended in RPMI 1640 medium to obtain a final concentration of 5 × 10^3^ CFU/mL. The evaluation of the antifungal activity of hydrazone derivatives was performed against *C. albicans*, *C. dubliniensis*, *C. glabrata*, *C. parapsilosis* and *C. tropicalis* cultured in 96-well microplates at different concentrations (0.06 μg/mL to 32 μg/mL) at 37 °C for 24 h. Growth and sterility controls were also used. On the other hand, a positive control was also realized with fluconazole (0.5 μg/mL and 0.06 μg/mL). Fungal growth was determined in colorimetric assays (Alamarblue^TM^, Thermo Fisher Scientific Inc., Illkirch, France). Minimum IC (MIC_99_) was defined as the lowest concentration of hydrazone derivative that produces a reduction of 99% of the yeast growth compared to controls (in the absence of compound).

### 3.5. Cell Viability Assay

Human HEK293 cells (Human Embryonic Kidney 293) were purchased from the American Type Culture Collection (ATCC, Manassas, VA, USA) and were grown in triple flask to 95% confluence and resuspended for dispensing at 1.25 × 10^5^ cells/mL of DMEM, 10% FBS/Pen/Strep/l-glutamine. HEK293 cells were plated in 96-well microplates (5000 cells per well in 40 μL media (DMEM/10%FBS/Pen/Strep/l-glutamine)) before incubation in standard conditions (5% CO_2_; 95% humidity, 37 °C) for 24 h.

Subsequently, cells were overlaid with RPMI medium containing different concentrations of drugs (0.06 μg/mL to 32 μg/mL), and cell viability was determined using MTT reagent as per manufacturer’s recommendations.

### 3.6. Cancer Cell Proliferation Assay

Compounds **1c**, **1d**, **1i**, **1k** and **1l** were tested against a panel of 60 human cancer cell lines at the National Cancer Institute (NCI), Germantown, MD, USA [21]. The cytotoxicity studies were conducted using a 48-h exposure protocol using the sulforhodamine B (SRB) assay [22].

## 4. Conclusions

An attempt to isolate pyrazoles derivatives, by reacting benzylidenemalononitriles with hydrazines in refluxing ethanol, did not provide the target heterocyclic systems, as expected and as previously reported, but instead hydrazones whose structure was secured by both chemical evidence and X-ray studies. Indeed, the direct reaction of 3,4,5-trimethoxybenzaldehyde with 2,4-dichlorophenylhydrazine furnished the same hydrazone as that obtained from benzylidenemalononitrile with hydrazine. This study identified five hydrazones as promising antifungal agents (MIC_99_ values ranging from 16 to 32 µg/mL) against *Candida* spp. These compounds showed a CC_50_ (concentration at 50% cytotoxicity) value against HEK293 cells at >32 µg/mL and against NCI-60 cancer cell lines panel at >10 µM, demonstrating selective antifungal activity over cytotoxicity. Compared to known acylhydrazones and hydrazones previously reported in the literature, a selection of which is available in Figure 1, the newly synthesized hydrazones are positioned as very promising experimental molecules with antifungal activity on *Candida* spp.

## Figures and Tables

**Table 1:** MIC values of active hydrazones **1c, 1d**, **1i**, **1k** and **1l** on *Candida* spp.

		MIC Values (µg/mL) on *Candida* spp. ^[a,b,c,d]^							
Entry	Compound	*C. albicans* SC5314	*C. dubliniensis*	*C. glabrata*	*C. parapsilosis*	*C. tropicalis*	*C. albicans* (Mucoviscidosis)	*C. albicans* (R echinocandins)	*C. glabrata* (R azoles)
1	**1c**	32	>32 ^[e]^	32	32	>32	>32	>32	32
2	**1d**	32	>32	16	>32	>32	>32	>32	32
3	**1i**	32	>32	32	32	>32	>32	>32	32
4	**1k**	32	>32	16	32	32	>32	>32	>32
5	**1l**	32	>32	16	32	32	>32	>32	32
6	Fluconazole	0.5	0.5	0.5	0.5	0.5	0.5	0.5	>32

^[a]^ In vitro inhibition percentage of pathogens. ^[b]^ Values represent mean of three experiments. ^[c]^ Compounds were tested at 32, 16, 8, 4 and 1 µg/mL concentration. ^[d]^ MIC values given in the table correspond to MIC_99_ (total growth inhibition of *Candida* spp.). ^[e]^ MIC not calculated since total inhibition of *Candida* spp. was not obtained at 32 µg/mL.

## Data Availability

The data presented in this study are available on request from the corresponding author. Supplementary Materials include X-Ray crystallography and NCI-60 data.

## Supplementary Materials

The following are available online: full description of the crystal structure of **1e** and **1i**, and one-dose full graphs obtained for active molecules **1c**, **1d**, **1i**, **1k** and **1l** on NCI-60 cancer cell lines panel associated with this article.

## Sample Availability

Samples of the compounds **1a**–**o** are available from the authors.
