# Supplementary material for: Attempts to Access a Series of Pyrazoles Lead to New Hydrazones with Antifungal Potential against Candida species including Azole-Resistant Strains
Source: Molecules. 2021 Sep 27;26(19):5861. doi: 10.3390/molecules26195861 (PMC8512829; doi:10.3390/molecules26195861)
Supplement: Supplementary file 1 [file molecules-26-05861-s001.zip › molecules-1367454-supplementary.pdf]

Supplementary Materials

Attempts to Access a Series of Pyrazoles Lead to New Hydrazones with Antifungal Potential against Candida Species Including Azole-Resistant Strains

Georgiana Negru 1, Laure Kamus 2, Elena Bîcu 1,*, Sergiu Shova 3, Boualem Sendid 2, Faustine Dubar 2,* and Alina Ghinet 1,4,5,*

1 Faculty of Chemistry, "Alexandru Ioan Cuza" University of Iași, Bd. Carol I 11, 700506 Iași, Romania;
georgiana_16_95@yahoo.com
2 Inserm U1285, CHU Lille, UGSF UMR CNRS 8576, Glycobiology in Fungal and Clinical Applications,
Unité de Glycobiologie Structurale et Fonctionnelle, University of Lille, 59000 Lille, France;
laurekamus@aol.com (L.K.); boualem.sendid@univ-lille.fr (B.S.)
3 "Petru Poni" Institute of Macromolecular Chemistry, 700487 Iasi, Romania;
shova@icmpp.ro
4 Laboratory of Sustainable Chemistry and Health, Health and Environment Department, Junia, 59000 Lille, France
5 Inserm, CHU Lille, Institut Pasteur Lille, U1167—RID-AGE—Facteurs de Risque et Déterminants
Moléculaires des Maladies Liées au Vieillissement, University of Lille, 59000 Lille, France
* Correspondence: elena@uaic.ro (E.B.); faustine.dubar@univ-lille.fr (F.D.); alina.ghinet@junia.com (A.G.)

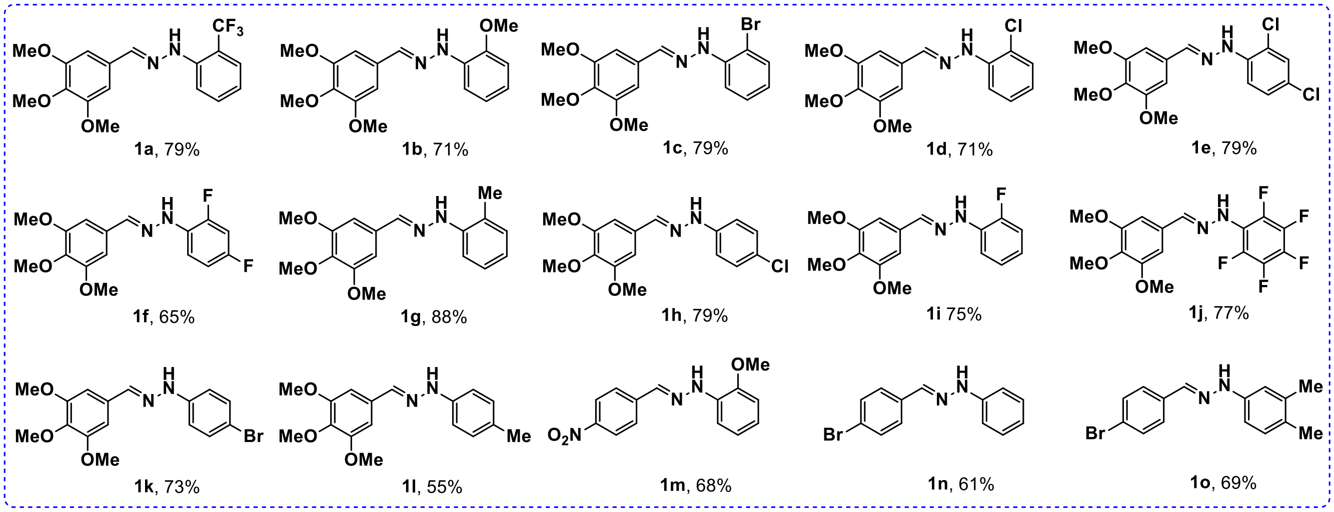

Figure S1. Structure of target hydrazones **1a-o**

X-ray crystallography

X-ray diffraction measurements for **1e** and **1i** were carried out with a Rigaku Oxford-Diffraction XCALIBUR E CCD diffractometer equipped with graphite-monochromated MoKα radiation. The unit cell determination and data integration were carried out using the CrysAlis package of Oxford Diffraction.[1] The structures were solved by Intrinsic Phasing using Olex2[2] software with the SHELXT[3] structure solution program and refined by full-matrix least-squares on F^2 with SHELXL-2015[4] using an anisotropic model for non-hydrogen atoms. All H atoms attached to carbon were introduced in idealized positions (d_{CH} = 0.96 Å) using the riding model with their isotropic displacement parameters fixed at 120% of their riding atom. The positions of H atoms for NH groups were determined from Fourier synthesis maps and verified through the hydrogen bonds parameters. Table S1 provides a summary of the crystallographic data together with refinement details for compounds. The geometric parameters are summarized in Table S2. The supplementary crystallographic data can be obtained free of charge via www.ccdc.cam.ac.uk/conts/retrieving.html (or from the Cambridge Crystallographic Data Centre, 12 Union Road, Cambridge CB2 1EZ, UK; fax: (+44) 1223–336-033; or deposit@ccdc.ca.ac.uk).

Table S1. Crystal data and details of data collection

Parameter	**1e**	**1i**
empirical formula	$C_{16}H_{16}Cl_2N_2O_3$	$C_{16}H_{17}FN_2O_3$
Fw	355.21	304.31
space group	$P2_1/c$	$P2_1/c$
a [Å]	15.4951(11)	14.3054(9)
b [Å]	7.8815(5)	7.8734(4)
c [Å]	15.5152(12)	15.509(2)
α [°]	90	90
β [°]	117.006(9)	117.591(6)
γ [°]	90	90
V [Å^3]	1688.2(2)	1548.1(2)
Z	4	4
r_{calcd} [g·cm^{-3}]	1.398	1.306
Crystal size [mm]	0.25 × 0.20 × 0.10	0.30 × 0.20 × 0.10
T [K]	293	293
μ [mm^{-1}]	0.400	0.099
2Θ range [°]	5.258 to 50.054	3.212 to 50.054
Reflections collected	7201	8594
Independent reflections	2989[R_{int}=0.0321]	2699[R_{int}=0.0257]
Data/restraints/paramet	2989/0/211	2699/0/202
R_1[a]	0.0470	0.0499
wR_2[b]	0.1121	0.1245
GOF[c]	1.020	1.020
Largest diff. peak/hole	0.25/-0.30	0.20/-0.18
CCDC No.	2097685	2097687

[a] $R_1 = \Sigma||F_o| - |F_c||/\Sigma|F_o|$. [b] $wR_2 = \{\Sigma[w(F_o^2 - F_c^2)^2]/\Sigma[w(F_o^2)^2]\}^{1/2}$. [c] $GOF = \{\Sigma[w(F_o^2 - F_c^2)^2]/(n - p)\}^{1/2}$, where *n* is the number of reflections and *p* is the total number of parameters refined.

Table S2. Bond distances (Å) and angles(°).

Compound **1e**.

O1-C9	1.418(3)
O2-C4	1.377(3)
O2-C8	1.429(3)
O3-C3	1.368(3)
O3-C7	1.427(3)
N1-N2	1.370(3)
N1-C10	1.274(3)
N2-C11	1.387(3)
C1-C2	1.392(3)
C1-C6	1.396(3)
C1-C10	1.458(3)
C2-C3	1.379(3)
C3-C4	1.395(3)
C4-C5	1.385(3)

C5-C6	1.383(3)
C11-C12	1.390(3)
C11-C16	1.388(3)
C12-C13	1.384(3)
C13-C14	1.370(4)
C14-C15	1.369(4)
C15-C16	1.382(3)

O3-C3-C2	124.9(2)
O3-C3-C4	114.7(2)
C2-C3-C4	120.4(2)
O2-C4-C3	120.8(2)
O2-C4-C5	119.4(2)
C5-C4-C3	119.6(2)
O1-C5-C4	114.6(2)
O1-C5-C6	124.9(2)
C6-C5-C4	120.5(2)
C5-C6-C1	119.7(2)
N1-C10-C1	122.4(2)
N2-C11-C12	120.3(2)
N2-C11-C16	122.0(2)
C16-C11-C12	117.7(2)
C11-C12-Cl1	119.5(2)
C13-C12-Cl1	118.8(2)
C13-C12-C11	121.7(3)
C14-C13-C12	119.0(3)
C13-C14-Cl2	119.3(3)
C15-C14-Cl2	120.0(3)
C15-C14-C13	120.7(3)
C14-C15-C16	120.1(3)
C15-C16-C11	120.7(3)

Compound **1i**.

F1-C12	1.359(3)
O1-C5	1.369(2)
O1-C9	1.426(3)
O2-C4	1.378(2)
O2-C8	1.425(3)
O3-C3	1.363(2)
O3-C7	1.424(3)
N1-N2	1.366(2)
N1-C10	1.276(2)
N2-C11	1.381(3)
C1-C2	1.392(3)
C1-C6	1.391(3)
C1-C10	1.459(3)
C2-C3	1.379(3)
C3-C4	1.398(3)

C4-C5	1.389(3)
C5-C6	1.381(3)
C11-C12	1.377(3)
C11-C16	1.385(3)
C12-C13	1.373(4)
C13-C14	1.362(5)
C14-C15	1.372(5)
C15-C16	1.383(4)

C6-C1-C10	117.98(18)
C3-C2-C1	120.29(19)
O3-C3-C2	125.13(19)
O3-C3-C4	115.09(18)
C2-C3-C4	119.77(18)
O2-C4-C3	120.31(18)
O2-C4-C5	119.73(19)
C5-C4-C3	119.71(19)
O1-C5-C4	114.65(19)
O1-C5-C6	124.94(19)
C6-C5-C4	120.41(19)
C5-C6-C1	119.81(19)
N1-C10-C1	122.41(19)
N2-C11-C16	123.4(2)
C12-C11-N2	119.4(2)
C12-C11-C16	117.2(2)
F1-C12-C11	117.5(2)
F1-C12-C13	119.4(3)
C13-C12-C11	123.1(3)
C14-C13-C12	118.4(3)
C13-C14-C15	120.6(3)
C14-C15-C16	120.3(3)
C15-C16-C11	120.3(3)

The crystal is built-up from two-dimensional wave-like layers formed through the packing of supramolecular chains developing parallel to 110 plane. A partial diagram of the crystal packing viewed along *a* axis is shown in Figure S2.

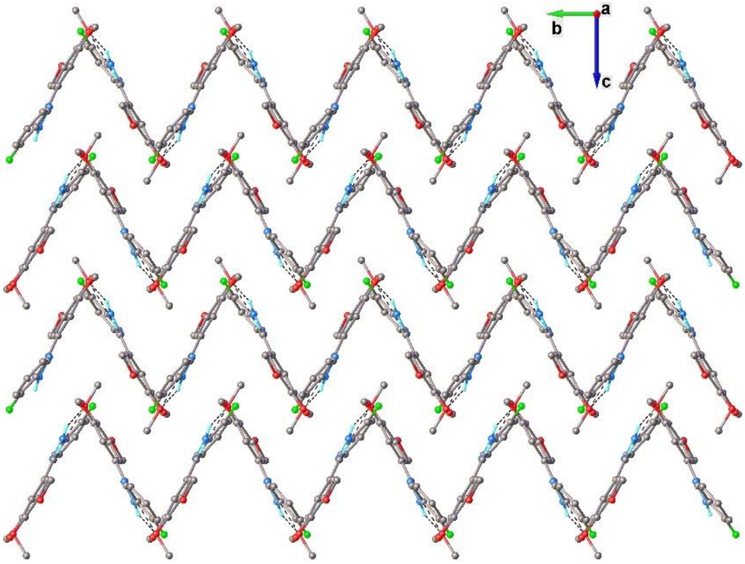

Figure S2. View of the crystal packing for **1i** along *a* axis.

National Cancer Institute (NCI) - One Dose Mean Graph for hydrazones **1c**, **1d**, **1i**, **1k** and **1l**

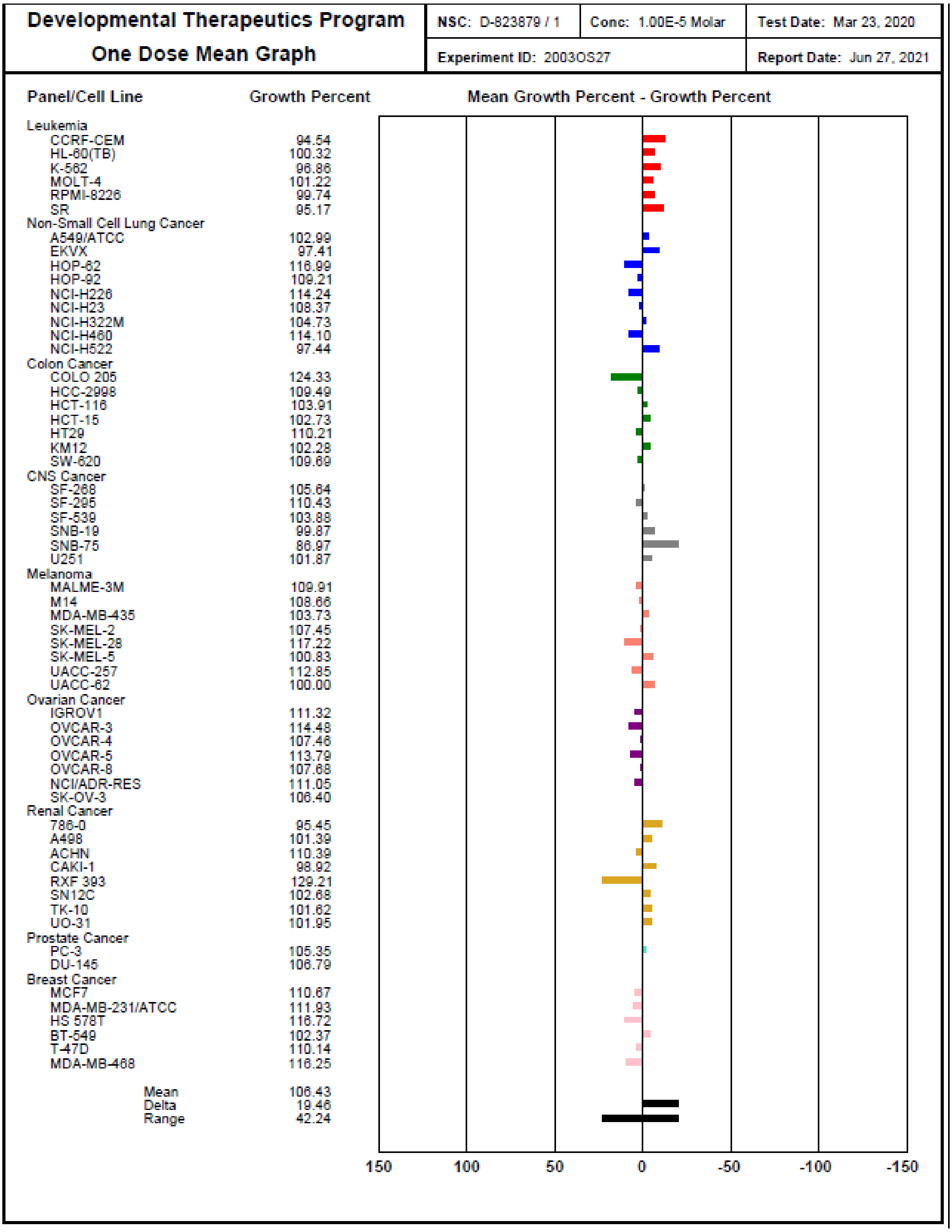

Developmental Therapeutics Program One Dose Mean Graph	NSC: D-824644 / 1	Conc: 1.00E-5 Molar	Test Date: Aug 17, 2020
	Experiment ID: 2008OS33		Report Date: Jul 21, 2021

Panel/Cell Line	Growth Percent
**Leukemia**	
CCRF-CEM	83.64
HL-60(TB)	82.85
K-562	66.71
MOLT-4	99.98
RPMI-8226	82.95
SR	88.13
**Non-Small Cell Lung Cancer**	
A549/ATCC	82.44
EKVX	82.72
HOP-62	96.65
HOP-92	58.63
NCI-H226	91.30
NCI-H23	88.38
NCI-H322M	90.54
NCI-H460	94.53
NCI-H522	75.12
**Colon Cancer**	
COLO 205	104.64
HCC-2998	107.83
HCT-116	76.96
HCT-15	70.23
HT29	106.18
KM12	77.06
SW-620	87.51
**CNS Cancer**	
SF-268	96.13
SF-295	79.30
SF-539	98.20
SNB-19	90.53
SNB-75	98.60
U251	86.46
**Melanoma**	
LOX IMVI	67.48
MALME-3M	112.69
M14	91.94
MDA-MB-435	88.64
SK-MEL-2	103.78
SK-MEL-28	105.57
SK-MEL-5	96.47
UACC-257	118.67
UACC-62	78.65
**Ovarian Cancer**	
IGROV1	80.14
OVCAR-3	93.43
OVCAR-4	77.54
OVCAR-5	94.31
OVCAR-8	98.90
NCI/ADR-RES	100.29
SK-OV-3	109.02
**Renal Cancer**	
786-0	95.27
A498	92.01
ACHN	79.98
CAKI-1	56.10
RXF 393	102.58
SN12C	87.15
TK-10	109.49
UO-31	69.14
**Prostate Cancer**	
PC-3	55.03
DU-145	96.49
**Breast Cancer**	
MCF7	75.02
MDA-MB-231/ATCC	83.53
HS 578T	100.55
BT-549	99.95
T-47D	85.12
Mean	88.97
Delta	33.94
Range	63.64

Mean Growth Percent - Growth Percent

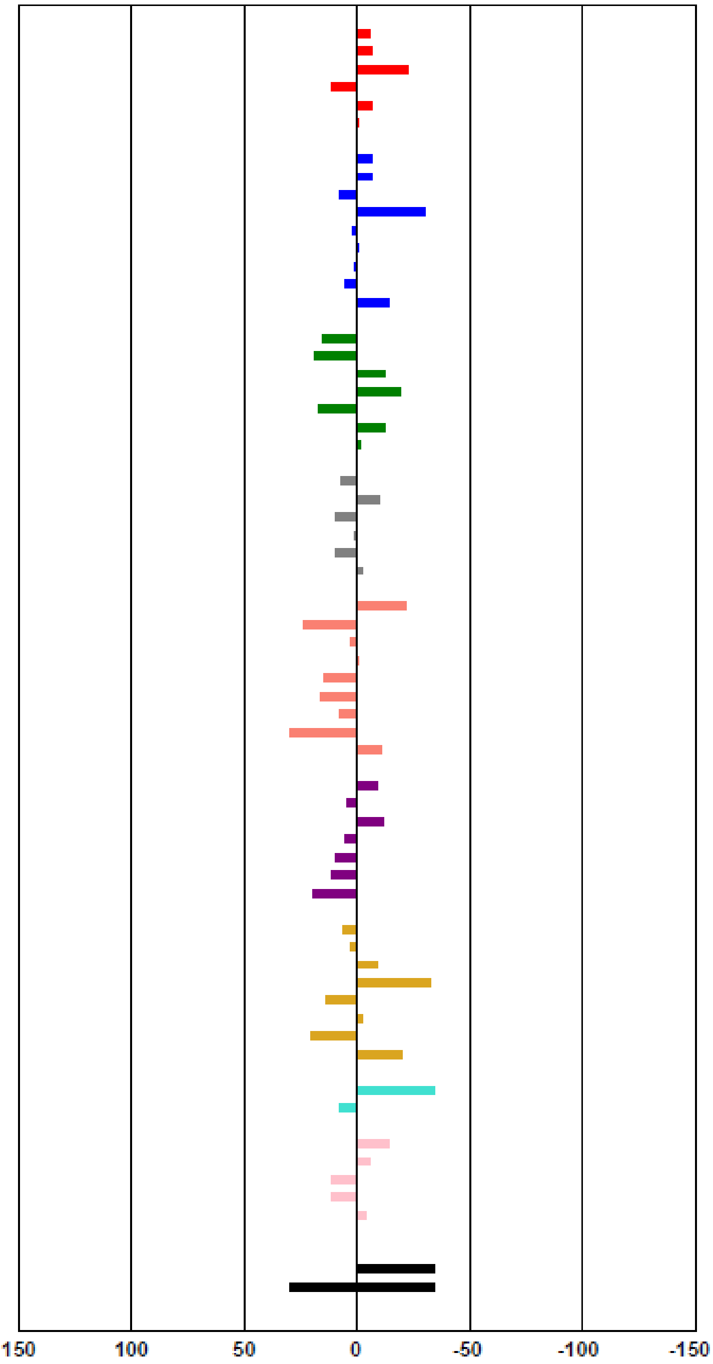

**Developmental Therapeutics Program One Dose Mean Graph**	**NSC:** D-816693 / 1	**Conc:** 1.00E-5 Molar	**Test Date:** Jul 15, 2019
	**Experiment ID:** 1907OS39		**Report Date:** Jul 21, 2021

Panel/Cell Line	Growth Percent
Leukemia	
CCRF-CEM	75.17
HL-60(TB)	90.06
K-562	48.52
MOLT-4	83.86
RPMI-8226	83.75
SR	90.95
Non-Small Cell Lung Cancer	
A549/ATCC	95.53
EKVX	87.40
HOP-62	94.35
HOP-92	99.12
NCI-H226	95.55
NCI-H23	100.68
NCI-H322M	91.26
NCI-H460	101.19
NCI-H522	68.69
Colon Cancer	
COLO 205	108.91
HCC-2998	107.87
HCT-116	84.49
HCT-15	89.24
HT29	93.55
KM12	86.33
SW-620	93.62
CNS Cancer	
SF-268	83.20
SF-295	98.09
SF-539	99.27
SNB-19	98.29
SNB-75	102.52
U251	93.28
Melanoma	
LOX IMVI	65.74
MALME-3M	99.03
M14	96.52
MDA-MB-435	94.32
SK-MEL-2	90.18
SK-MEL-28	109.62
SK-MEL-5	102.38
UACC-257	115.65
UACC-62	65.72
Ovarian Cancer	
IGROV1	86.12
OVCAR-3	108.04
OVCAR-4	92.30
OVCAR-5	94.64
OVCAR-8	92.03
NCI/ADR-RES	99.60
SK-OV-3	111.24
Renal Cancer	
786-0	90.37
A498	92.82
ACHN	81.31
CAKI-1	50.76
RXF 393	123.35
SN12C	97.10
TK-10	157.05
UO-31	68.83
Prostate Cancer	
PC-3	88.72
DU-145	106.36
Breast Cancer	
MCF7	78.26
MDA-MB-231/ATCC	81.46
HS 578T	94.89
BT-549	95.99
T-47D	67.69
MDA-MB-468	97.04
Mean	92.33
Delta	43.81
Range	108.53

Mean Growth Percent - Growth Percent

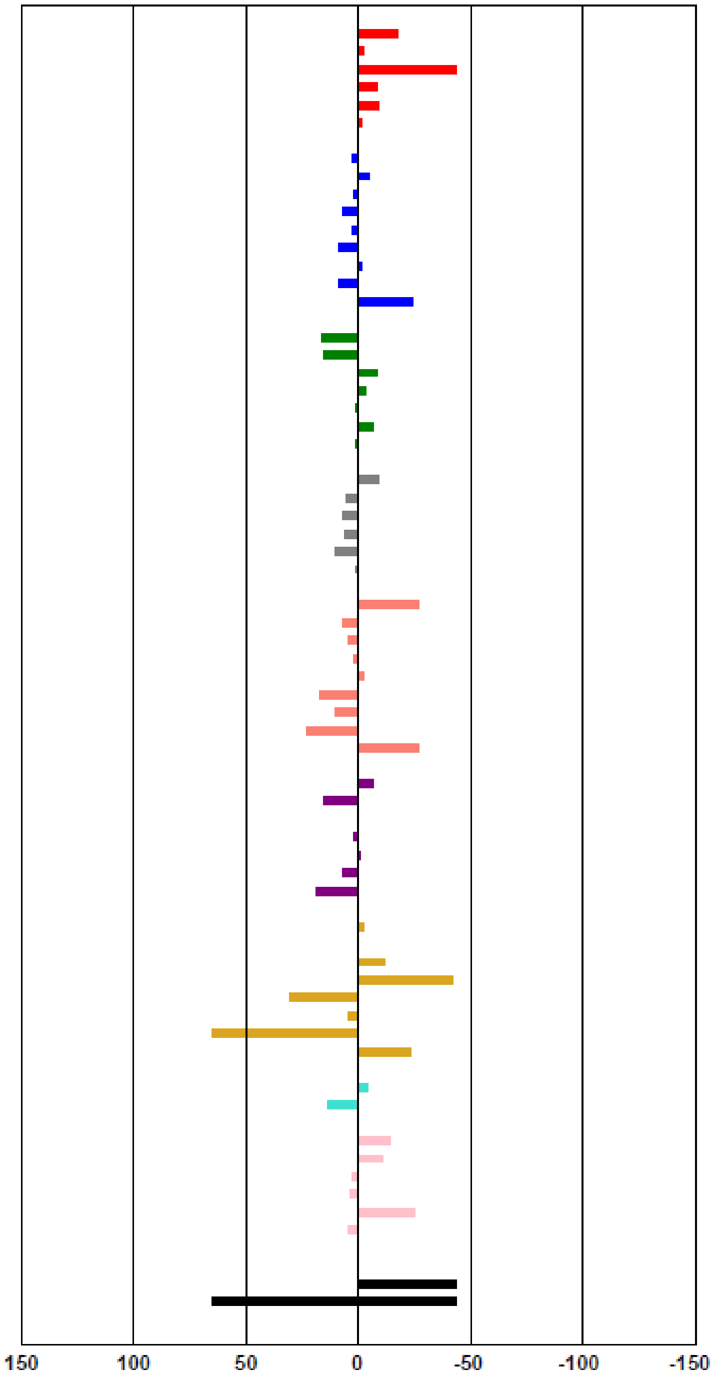

Developmental Therapeutics Program One Dose Mean Graph	NSC: D-816692 / 1	Conc: 1.00E-5 Molar	Test Date: Jul 15, 2019
	Experiment ID: 1907OS39		Report Date: Jul 21, 2021

Panel/Cell Line	Growth Percent
**Leukemia**	
CCRF-CEM	31.13
HL-60(TB)	33.04
K-562	9.82
MOLT-4	44.12
RPMI-8226	58.37
SR	68.77
**Non-Small Cell Lung Cancer**	
A549/ATCC	54.94
EKVX	63.89
HOP-62	93.09
HOP-92	98.17
NCI-H226	80.08
NCI-H23	75.75
NCI-H322M	66.30
NCI-H460	31.08
NCI-H522	39.77
**Colon Cancer**	
COLO 205	86.94
HCC-2998	45.74
HCT-116	60.75
HCT-15	18.91
HT29	29.21
KM12	16.33
SW-620	46.85
**CNS Cancer**	
SF-268	52.45
SF-295	60.87
SF-539	63.53
SNB-19	91.62
SNB-75	87.51
U251	65.52
**Melanoma**	
LOX IMVI	13.54
MALME-3M	55.52
M14	18.37
MDA-MB-435	39.52
SK-MEL-2	72.97
SK-MEL-28	82.15
SK-MEL-5	85.01
UACC-257	84.63
UACC-62	36.77
**Ovarian Cancer**	
IGROV1	57.26
OVCAR-3	63.27
OVCAR-4	50.98
OVCAR-5	68.36
OVCAR-8	54.77
NCI/ADR-RES	57.23
SK-OV-3	85.17
**Renal Cancer**	
786-0	77.85
A498	60.03
ACHN	31.60
CAKI-1	10.17
RXF 393	101.76
SN12C	69.73
TK-10	97.19
UO-31	34.98
**Prostate Cancer**	
PC-3	60.93
DU-145	63.10
**Breast Cancer**	
MCF7	50.96
MDA-MB-231/ATCC	47.49
HS 578T	79.98
BT-549	95.05
T-47D	30.76
MDA-MB-468	49.46
Mean	57.69
Delta	47.87
Range	91.94

Mean Growth Percent - Growth Percent

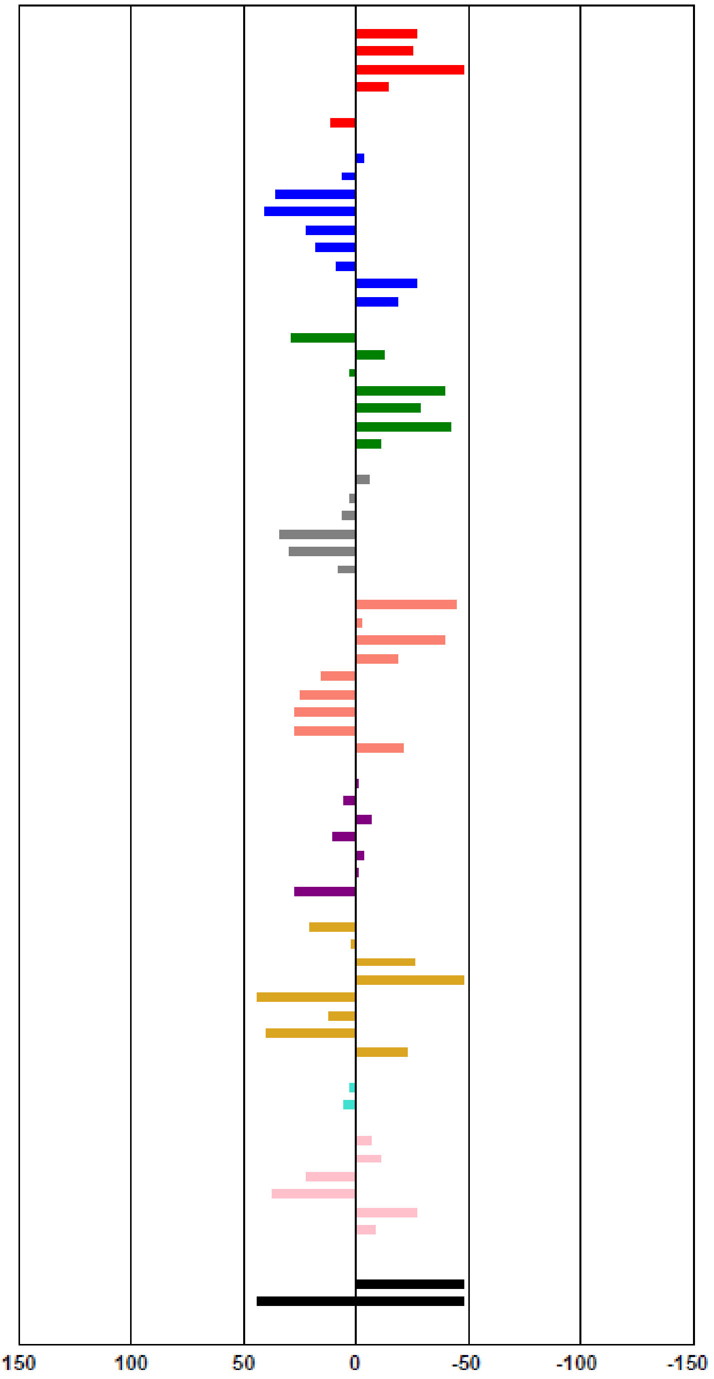

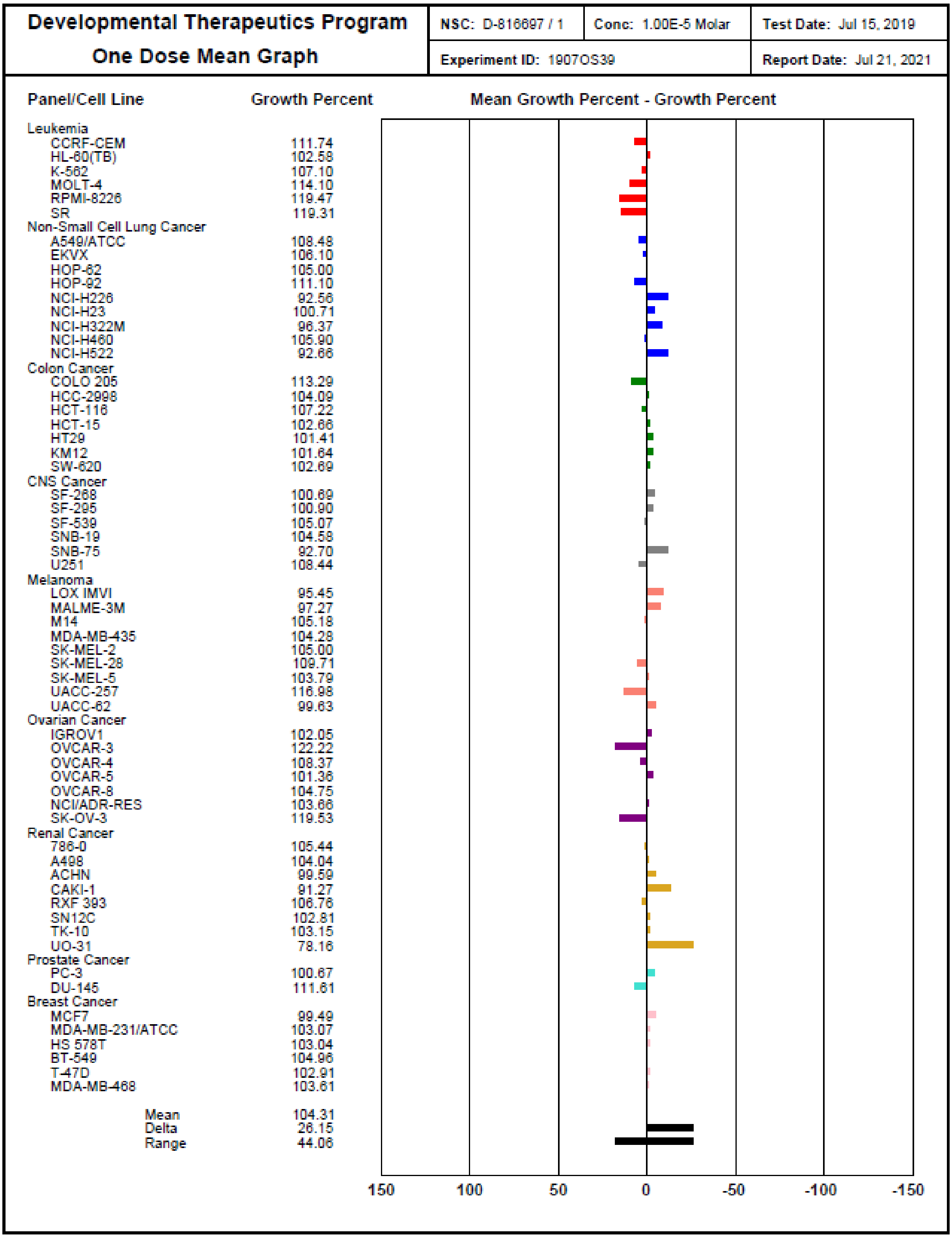

References

[1] Rigaku Oxford Diffraction, **2015**, CrysAlisPro Software system, version 1.171.38.46, Rigaku Corporation, Oxford, UK.

[2] Dolomanov, O. V.; Bourhis, L. J.; Gildea, R. J.; Howard, J. A. K.; Puschmann, H. *J. Appl. Crystallogr*. **2009**, *42*, 339.

[3] Sheldrick, G. *Acta Cryst. A* **2015**, *71*, 3.

[4] Sheldrick, G. *Acta Cryst. C* **2015**, *71*, 3.
